# Increased Tc17 cell levels and imbalance of naïve/effector immune response in Parkinson’s disease patients in a two-year follow-up: a case control study

**DOI:** 10.1186/s12967-021-03055-2

**Published:** 2021-09-06

**Authors:** Diana D. Álvarez-Luquín, Adrián Guevara-Salinas, Asiel Arce-Sillas, Raquel Espinosa-Cárdenas, Jaquelín Leyva-Hernández, Esteban U. Montes-Moratilla, Laura Adalid-Peralta

**Affiliations:** Unidad Periférica Para El Estudio de La Neuroinflamación en Patologías Neurológicas del Instituto de Investigaciones Biomédicas en El Instituto Nacional de Neurología Y Neurocirugía, Insurgentes Sur 3877, La Fama, 14269 Ciudad de México, México

**Keywords:** Immune response, Parkinson’s disease, Untreated patients, Immunophenotyping, Flow cytometry

## Abstract

**Background:**

Neuroinflammation has been proved to play a role in dopaminergic neuronal death in Parkinson’s disease (PD). This link highlights the relevance of the immune response in the progression of the disease. However, little is known about the impact of peripheral immune response on the disease. This study is aimed to evaluate how immune cell populations change in untreated PD patients followed-up for 2 years.

**Methods:**

Thirty-two patients with no previous treatment (PD-0 yr) and twenty-two healthy subjects (controls) were included in the study. PD patients were sampled 1 and 2 years after the start of the treatment. CD4 T cells (naïve/central memory, effector, and activated), CD8 T cells (activated, central memory, effector memory, NKT, Tc1, Tc2, and Tc17), and B cells (activated, plasma, and Lip-AP) were characterized by flow cytometry.

**Results:**

We observed decreased levels of naïve/central memory CD4 and CD8 T cells, Tc1, Tc2, NKT, and plasma cells, and increased levels of effector T cells, activated T cells, and Tc17.

**Conclusions:**

PD patients treated for 2 years showed an imbalance in the naive/effector immune response. Naïve and effector cell levels were associated with clinical deterioration. These populations are also correlated to aging. On the other hand, higher Tc17 levels suggest an increased inflammatory response, which may impact the progression of the disease. Our results highlight the relevant effect of treatment on the immune response, which could improve our management of the disease.

**Supplementary Information:**

The online version contains supplementary material available at 10.1186/s12967-021-03055-2.

## Introduction

Parkinson’s disease (PD), the second leading neurodegenerative disease in the world, is characterized by the death of dopaminergic neurons. PD affects nearly 1% of the world population aged 60 and over [1]. Recent data indicate an increase of 21.7% in the global prevalence of the disease in the period 1990–2016. PD prevalence is expected to double by 2030, mainly due to population aging [2, 3].

PD is characterized by symptoms like bradykinesia, resting tremor, and rigidity [4]. Several scales have been proposed to assess PD progression and severity, focusing on both motor and non-motor symptoms; these include the Hoehn and Yahr (H&Y) scale, the Movement Disorder Society-sponsored revision of the unified Parkinson’s disease rating scale (MDS-UPDRS), Becks Inventory, and Schwab and England [5, 6].

The current treatment is aimed to alleviate the most conspicuous symptoms of the disease. Levodopa is the most effective drug to treat motor symptoms in PD patients, but dopaminergic agonists are also used to replace dopamine in early-onset PD cases in young individuals. Various studies have shown the effect of activating dopamine receptors on immune cells by stimulating them with dopamine and dopamine agonists [7, 8]. While there is evidence that levodopa and dopamine agonists can affect the immune system, the effects of these drugs on immune cell populations in an early stage are not completely understood.

A few works have characterized the immune cell populations after PD treatment. Koziorowski [9] found increased levels of TNF-α and the N-terminal pro-C-type natriuretic peptide (NT-proCNP) in serum samples from PD patients treated with levodopa, with respect to a control group [9]. Other research teams have found decreased levels of total lymphocytes (CD3+) [10], CD4+ CD8− [10], CD4+ CD25+ [10], and CD4+CD25^HI^ cells [10], IL-4 producing T cells [10], pDCs (CD123+ HLA-DR+) [11], and mDCs (CD11c+ HLA-DR+) [11]. Moreover, increased counts of CD4^bright^ CD8^dull^ cells have been reported [10]. A study by our group comparing patients treated with levodopa and pramipexole with the same group of patients before treatment found a decrease in Bregs (CD19+CD5+CD1d+FOXP3+IL-10) and plasma cells (CD19+CD138−IL-10+), as well as increased levels of active Tregs (CD4+CD45RO+FOXP3HI), functional Tregs (CD4+CD25HIFOXP3+CD127−/LOW), Tr1 (CD4+CD25HIIL-10+) classical monocytes (CD16−CD14HIIL-10+), and functional Bregs (CD19+CD24+CD38+IL-10+) [12].

Other works have described the immune populations in treated PD patients. However, it should be noted that patients in most studies were not segregated by the treatment they received. PD treated patients have shown decreased levels of naïve T cells, Tregs, dendritic cells, Th1, Th2, and Th17 cells, as well as increased counts of effector T, memory T, Th1, Th17, and NK cells, along with classical monocytes [13–19].

These reports expose the complex response of the immune system to the treatment in PD patients. Unfortunately, few longitudinal studies have evaluated the effect of levodopa and pramipexole on immunity.

The link between a pro-inflammatory component and PD pathogenesis is clear, but the exact role of immune cell populations in the physiopathology of the disease needs to be defined. Few studies have assessed the impact of PD treatment on the immune populations, and even fewer followed-up those populations. Thus, considering the relevance of inflammation in PD, the changes in the peripheral immune response in PD patients treated with levodopa and/or pramipexole were herein followed-up over a 2-year period.

## Material and methods

All recruited patients attended the Instituto Nacional de Neurología y Neurocirugía (INNN) and agreed to participate in this study by signing an informed consent letter. In this study, patients diagnosed with idiopathic PD were followed-up. Thirty-two patients with no previous treatment (PD-0 yr) and twenty-two healthy subjects (controls) were included. After 1 year of treatment (PD-1 yr), a second blood sample was collected from 22 patients and 19 controls. 2 years after recruitment and PD treatment start (PD-2 yr), a third blood sample was obtained from 10 patients and 12 controls. The patients were evaluated regularly by a neurologist, who adjusted the treatment as required for the disease to be in control according to international guidelines [20, 21]. In this protocol, PD patients treated with levodopa, pramipexole, or a levodopa/pramipexole combination were followed up (PD-1 yr and PD-2 yr). PD patients receiving any other treatment were excluded [20, 21]. After 2 years, about 60% of patients had dropped out of the study, mostly due to lack of therapeutic compliance.

The protocol was approved by the INNN Ethics Committee (Permit No. 95/14) and was conducted in accordance with the Helsinki Declaration. All diagnoses were performed by an expert neurologist at the INNN, following the UK Parkinson’s Disease Society Brain Bank (UK PDSBB) diagnosis criteria. The MDS-UPDRs, H&Y, Schwab and England, and Beck depression questionnaires were applied to comprehensively evaluate the patients clinical and emotional status.

### Samples

Twenty milliliters of peripheral blood were collected from each patient and control subject in tubes containing acid-citrate-dextrose (ACD) (Vacutainer ACD, BD Franklin Lakes, NJ, USA). Blood samples were centrifuged to obtain plasma, and the cells were resuspended in sterile phosphate buffer solution (PBS) 1×. The samples were diluted 1/2 with sterile PBS 1×. Peripheral blood mononuclear cells (PBMCs) were separated by density gradient using Ficoll-Hypaque (Sigma Aldrich, Little Chalfont, UK). The samples were centrifuged for 30 min at 1800 rpm, without brake. Subsequently, the PBMCs were separated and washed twice with PBS 1×, centrifuging for 10 min at 1600 rpm. Finally, the cells were resuspended in 1 mL of PBS 1 × and counted in a Neubauer chamber using Trypan Blue staining. Only samples with at least 95% of cellular viability were used to characterize cellular phenotypes by flow cytometry.

### Cell population labeling for flow cytometry analysis

For each phenotype and isotype, 10^6^ PBMCs in a total volume of 50 µL were stained and analyzed. For intracellular labeling, PBMCs were incubated with brefeldin (10 µg/mL) for 4 h at 37 °C and 5% CO_2_ before labeling. Cytokine production was assayed in unstimulated PBMCs, since it has been reported that cytokine induction heavily depends on the stimulus used [22]; additionally, the analysis of unstimulated PBMCs has been reported previously [23–25]. Thus, only spontaneous cytokine production was considered in this study. Thereafter, antibodies for extracellular labeling were added and incubated for 30 min at 4 °C. PBMCs were washed with PBS supplemented with bovine serum albumin 5% and fetal bovine serum 1%. Then, PBMCs were permeabilized by adding 300 µL of Fixation and Permeabilization Buffers (eBioscience, Waltham, MA, USA). PBMCs were then incubated at 4 °C for 2 h, washed with Permeabilization Buffer (eBioscience), blocked with 20 µL of 10% rat serum solution, and incubated again for 1 h at 4 °C. Intracellular antibodies were added with no previous wash, and the PBMCs were incubated for 30 min at 4 °C. Finally, PBMCs were washed with Permeabilization Buffer and fixed with 200 µL of 2% paraformaldehyde. The antibodies used and their isotypes are shown in the Additional File 1: Table S1. FMO and isotype controls were used to discriminate positive from negative cells. Labeled cells were read in an Attune Acoustic Focusing Cytometer (Applied Biosystems, Waltham, MA, USA), and analyzed with the Attune Cytometric Software v.1.2.5. All phenotypes analyzed in this study are shown in the Additional File 2: Table S2. The strategy used in the flow cytometry analysis is shown in the Additional File 3: Figure S1.

### Statistical analysis

Statistical charts and analyses were prepared and conducted with Prism 8 (GraphPad Software, San Diego, CA). A Shapiro–Wilk test was performed to determine data normality. According to the normality test, either a student’s *t*-test or a Mann–Whitney U-test were used to compare patient and control groups. It should be noted that control samples taken at different times were pooled. Data of patients at different times (PD-0 yr, PD-1 yr, and PD-2 yr) were compared by a paired student’s *t*-test or a Wilcoxon signed-rank test, according to normality. Correlations between changes in the immune response and various patient variables (H&Y score, MDS-UPDRS, Becks Inventory, Schwab and England, age, and treatment (levodopa equivalent doses)) were performed. Correlation analyses were performed with either the Spearman or Pearson tests, according to the results of normality test.

## Results

### Clinical features of patients and controls

To evaluate how the immune response evolves in patients treated with levodopa and/or pramipexol for the first time, we determined immune cell phenotypes over a 2-year period. Untreated patients (PD-0 yr) had a mean H&Y and MDS-UPDRS score of 2.17 and 51.59, respectively, indicating a mild motor stage of the disease (Table 1). The symptoms were reported to appear 2 years before the first consultation in average. No significant differences were observed in body mass index of patients compared with controls. The most frequent comorbidity in the patient group was high blood pressure (HBP), present in 33.3% of patients.

**Table 1:** Clinical features of PD patients and healthy controls

	Ctrl-0 yr	PD-0 yr	Ctrl-1 yr	PD-1 yr	Ctrl-2 yr	PD-2 yr	Ctrl-0 yr vs PD-0 yr *P-value*	Ctrl-1 yr vs PD-1 yr *P-value*	Ctrl-2 yr vs PD-2 yr *P-value*
N	22	32	19	22	12	10	–	–	–
Age (years)^¢^	55.59 ± 10.22	60.81 ± 10.23	54.37 ± 8.01	61.95 ± 11.95	56.42 ± 10.35	57.70 ± 11.04	0.072	0.027*	0.662
Male: female ratio^	54**:**46	56**:**44	58:42	67:33	58:42	50:50	–	–	–
Body mass index^¢^	26.85 ± 3.89	27.52 ± 4.54	27.13 ± 3.62	27.14 ± 4.26	27.17 ± 4.38	26.87 ± 5.68	0.549	0.952	0.974
Symptom duration (years) ^¢^	NA	2 ± 1.17	NA	2.64 ± 1.39	NA	4.56 ± 1.59	–	–	–
Hoehn and Yahr score^€^	NA	2.00(1–3)	NA	1.25(0–3)	NA	2.25 (0–3)	–	–	–
Total MDS-UPDRS^¢^	2.77 ± 4.05	51.59 ± 25.13	0.89 ± 1.85	39.57 ± 24.47	1.42 ± 2.87	39.90 ± 27.24	< 0.0001*	< 0.0001*	< 0.0001*
MDS-UPDRS I^¢^	0.72 ± 0.98	2.50 ± 1.60	0.21 ± 0.42	1.81 ± 1.54	0.50 ± 0.80	2.27 ± 2.21	< 0.0001*	< 0.0001*	0.0062
MDS-UPDRS II^¢^	0.32 ± 0.89	13.28 ± 6.56	0.10 ± 0.31	10.62 ± 6.59	0.50 ± 1.17	9.40 ± 7.49	< 0.0001*	< 0.0001*	< 0.0001*
MDS-UPDRS III^¢^	1.72 ± 3.71	35.68 ± 18.29	0.58 ± 1.64	26.05 ± 16.69	0.42 ± 1.16	24.50 ± 19.06	< 0.0001*	< 0.0001*	< 0.0001*
Schwab and England scale^¢^	99.54 ± 2.13	76.59 ± 22.55	100 ± 0	86 ± 11.42	100 ± 0	86.37 ± 12.25	< 0.0001*	< 0.0001*	< 0.0001*
Beck’s Depression Inventory^¢^	5.54 ± 6.24	11.50 ± 8.00	2.42 ± 3.04	9.43 ± 7.41	5.83 ± 6.63	11.90 ± 10.90	0.003*	0.0013*	0.108
Comorbidities									
HBP	6.67%	33.3%	4.54%	45%	8.3%	40%	–	–	–
T2D	0%	10%	4.54%	9%	8.3%	10%	–	–	–
Hypercholesterolemia	0%	6.67%	0%	4.54%	0%	0%	–	–	–
Dyslipidemia	0%	6.67%	4.54%	4.54%	0%	10%	–	–	–

High blood pressure (HBP) Type 2 diabetes (T2D)

^data are reported as the percentage of subjects

^¢^data are reported as mean ± SD

^€^data are reported as median (range)

*P ≤ 0.05 was considered as significant

**Fig. 1:**
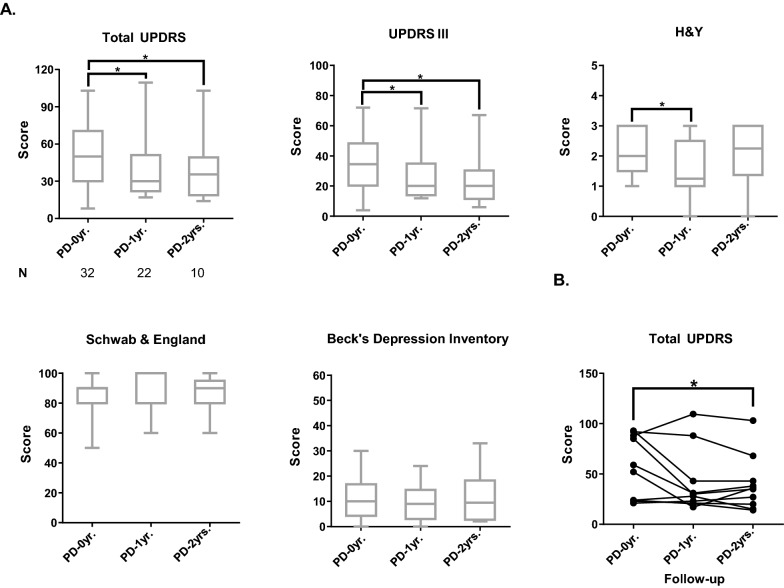
A clinical description of PD patients. **A** Score in clinical scales like MDS-UPDRS, H&Y, Beck, and Shwab and England. **B** Changes in MDS-UPDRS score of the 10 followed-up patients. PD-0 yr (untreated Parkinson’s disease patients), PD-1 yr (patients treated for one year), PD-2 yr (patients treated for two years), MDS-UPDRS (Unified Parkinson’s Disease Rating Scale), H&Y (Hoehn & Yahr scale). (*) indicates changes between patients. *P* < 0.05 (*); *P* < 0.005 (**); *P* < 0.0005 (***); *P* < 0.00001 (****)

All patients included in this study were diagnosed for the first time and had not received any kind of PD treatment before recruitment. The progression of clinical evaluation of PD patients during the follow-up period is shown in Fig. 1. We observed that total MDS-UPDRS and MDS-UPDRS III scores were lower in the PD-1 yr group than in PD-0 yr (*P* = 0.026 and *P* = 0.048, respectively) and PD-2 yr with respect to PD-0 yr (*P* = 0.012 and *P* = 0.018, respectively) probably due to treatment (Fig. 1A). Also, as shown in Fig. 1A, the Hoehn and Yahr scale score decreased between PD-0 yr and PD-1 yr (*P* = 0.043). In contrast, Schwab and England and Beck’s Depression Inventory scores were similar in patients over time. Finally, the Total MDS-UPDRS score of patients followed-up for 2 years is shown in Fig. 1B. A good response to treatment was observed in all patients, as shown by the decreased MDS-UPDRS scale scores.

### Imbalance of naïve and effector/activated CD4 T cells in Parkinson’s disease patients

**Fig. 2:**
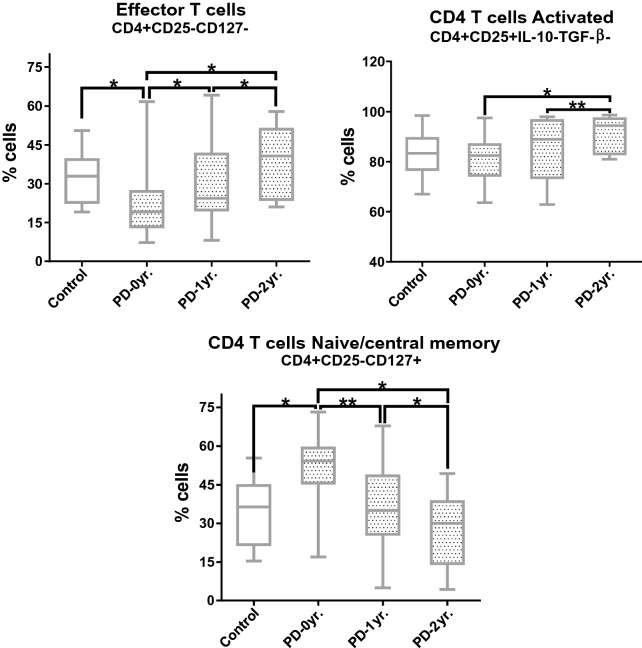
Changes in the levels of CD4 T in PD patients at inclusion time, one year, and two years after. PD-0 yr (untreated Parkinson’s disease patients) PD-1 yr (patients treated for one year), PD-2 yr (patients treated for two years). (*) indicates significant differences between patient groups. *P* < 0.05 (*); *P* < 0.005 (**)

To characterize the memory and effector response, we analyzed different phenotypes of CD4 T cells, including activated, naïve/central memory, and effector CD4 T cells. Total frequencies of CD4 + cells are reported in the Additional File 4: Table S3. We observed an increase in the levels of effector T cells (CD4 + CD25−CD127−) after 1 and 2 years (*P* = 0.029 and *P* = 0.048, respectively) with respect to PD-0 yr (Fig. 2). We also observed increased frequencies of these cells in PD-2 yr with respect to PD-1 yr (*P* = 0.027). A positive correlation was found between these cells and the H&Y scale score after two years (*r* = 0.8225, *P* = 0.019). Interestingly, we found a positive correlation between these cells and age (*r* = 0.599, *P* = 0.004) in the PD-1 yr group (Additional File 5: Table S4). On the other hand, an increase was observed in the levels of activated CD4 non-Tregs (CD4 + CD25 + IL-10-TGF-β −) in PD patients after two years with respect to PD-0 yr patients (*P* = 0.019), and with respect to PD-1 yr (*P* = 0.014). Moreover, we found a decrease in the frequencies of naïve/central memory CD4 T cells (CD4+CD25−CD127+) after one and two years with respect to PD-0 yr (*P* = 0.002 and *P* = 0.014, respectively), and between PD-2 yr and PD-1 yr (*P* = 0.019), likely related to the time of treatment (Fig. 2). Also, in PD-2yrs, we found a negative correlation with the score on the H&Y scale (*r* =  − 0.7979, *P* = 0.015). Remarkably, in PD-1 yr we found a negative correlation with age (*r* =  − 0.453, *P* = 0.039) (Additional File 5: Table S4).

### Tc17 cell levels are increased in Parkinson’s disease patients after 2 years of treatment

Considering that no previous studies have assessed changes in the CD8 population related to memory response, we determined the levels of activated, effector memory, and central memory CD8 T cells in PD patients. The total frequencies of CD8+ cells are reported in the Additional File 4: Table S3. The levels of activated-like (CD8+CD28+), effector memory (CD8+CCR7−CD45RO+), effector memory non-Tregs (CD8+CCR7−CD45RO+IL-10−), central memory non-Tregs (CD8+CCR7+CD45RO+), and Naïve CD8 non-Tregs (CD8+CCR7+CD45R−IL-10−) did not significantly change over time.

**Fig. 3:**
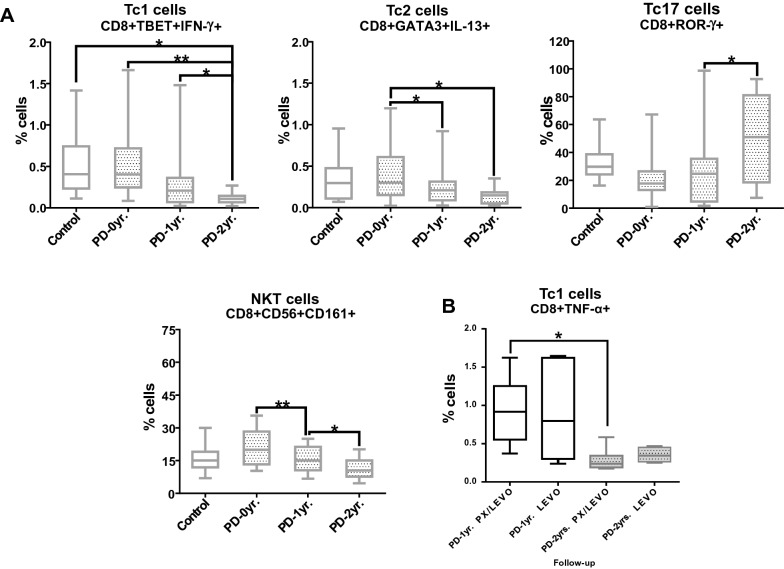
**A** Changes in the levels of CD8 T cells and NKT cells at inclusion time, 1 year and 2 years after treatment. **B** Changes in the levels of Tc1 cells in PD patients stratified by treatment. PD-0 yr (untreated Parkinson’s disease patients), PD-1 yr (patients treated for 1 year), PD-2 yr (patients treated for 2 years). (*) indicates significant differences between patient groups. *P* < 0.05 (*); *P* < 0.005 (**)

Similarly, no previous study has analyzed the changes in the levels of T cytotoxic cells (Tc1, Tc2, and Tc17) in PD patients. The analysis of Tc cells included two populations: one expressing the transcription factor only, and another expressing the transcription factor and cytokines (Fig. 3 and Additional File 6: Table S5). We found a decrease in the levels of Tc1 and Tc2 cells (Fig. 3A). The levels of Tc1 (CD8 + Tbet + IFN-γ +) cells decreased after 2 years with respect to PD-0 yr (*P* = 0.004) and PD-1 yr *(P* = 0.009). Additionally, the frequencies of Tc1 cells (CD8+TNF-α+) increased at PD-1 yr with respect to PD-0 yr (*P* = 0.011) but decreased at PD-2 yr with respect to PD-0 yr (*P* = 0.002) and PD-1 yr (*P* = 0.006).

To determine whether the immune response changed depending on PD treatment administered, we grouped PD patients into subjects receiving only levodopa and subjects receiving a levodopa/pramipexole combination. Only patients administered with a combined therapy showed decreased levels of Tc1 cells after 2 years of treatment. (Fig. 3B). Besides, we found a negative correlation at PD-2 yr between Tc1 cell levels and the H&Y scale score (r =  − 0.7746, *P* < 0.0001) in patients treated with levodopa only. The levels of Tc2 cells (CD8+GATA−3+IL-13+) were also decreased after one and 2 years with respect to PD-0 yr (*P* = 0.007 and *P* = 0.027, respectively). Additionally, we found a negative correlation at PD-2 yr between Tc2 cell levels and the H&Y scale score (*r* =  − 0.7746, *P* < 0.0001) in patients treated only with levodopa. On the other hand, the frequencies of Tc2 cells (CD8+IL-4+) decreased at PD-2 yr with respect to PD-1 yr (*P* = 0.027). Finally, the levels of Tc17 cells (CD8 + ROR-γ +) increased at PD-2 yr with respect to PD-1 yr (*P* = 0.039), while the levels of IL-17- nor IL-6- producing Tc17 cells changed significantly at any time point.

With regard to the subset of CD8 T cells, the levels of NKT cells and mucosal-associated-like invariant T cells (MAIT-like) were also analyzed. As shown in Fig. 3, a decreased on the percentage of NKT (CD8+CD56+CD161+) cells was observed after one and 2 years with respect to PD-0 yr (*P* = 0.002 and *P* = 0.016, respectively). On the other hand, we found a positive correlation between the levels of NKT cells with the MDS-UPDRS scale score after 1 year (*r* = 0.9276, *P* = 0.0222) in patients treated with levodopa only. No changes in the levels of MAIT cells (CD8+CD161^HI^) were observed at different times (PD-0 yr, 1.44 ± 0.79; PD-1 yr; 1.53 ± 0.62; PD-2 yr, 1.02 ± 0.74).

### Decreased levels of plasma and lip-AP B cells in treated Parkinson’s disease patients in the period under study

**Fig. 4:**
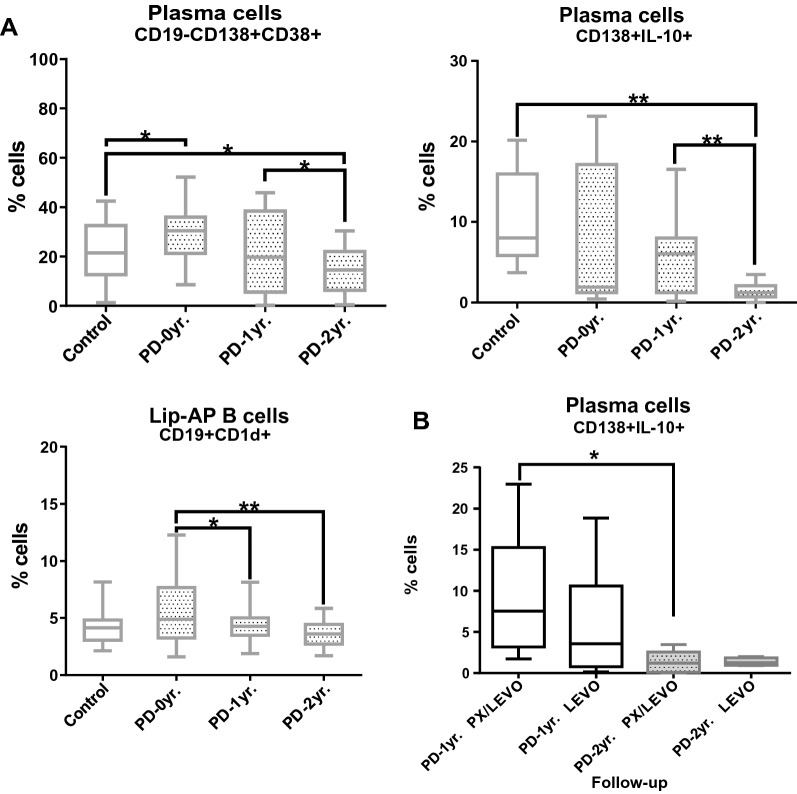
**A** Changes in the levels of B cells at inclusion time, 1 year and 2 years after. **B** Changes in the levels of plasma cells in PD patients stratified by treatment. PD-0 yr (untreated Parkinson’s disease patients), PD-1 yr (patients treated for 1 year), PD-2 yr (patients treated for 2 years). (*) indicates significant differences between patient groups. *P* < 0.05 (*); *P* < 0.005 (**); *P* < 0.0005 (***)

B lymphocytes have been poorly studied in PD patients as well. The subpopulations herein analyzed were activated B cells, IL-10+ plasma cells, and lip-AP B cells. The total frequencies of CD19 cells are reported in Additional File 4: Table S3. As shown in Fig. 4, we found a decrease in the number of plasma cells (CD19−CD138+CD38+) in the PD-2 yr group with respect to PD-1 yr (*P* = 0.048); interestingly, we found a positive correlation with age in the in PD-0 yr group (*r* = 0.461, *P* = 0.012) (Additional File 5: Table S4). Additionally, we found a decrease in the levels of IL-10+ plasma cells (CD138+IL-10+) in the PD-2 yr group with respect to PD-1 yr (*P* = 0.0078). On the other hand, we found a negative correlation between IL-10+ plasma cells with the H&Y scale score at PD-1 yr (*r* =  − 0.9487, *P* < 0.0001) in patients treated with levodopa in combination with pramipexole. Also, decreased levels of lip-AP B cells (CD19+CD1d+) were observed in the PD-1 yr and PD-2 yr groups with respect to PD-0 yr (*P* = 0.026 and *P* = 0.002, respectively), while no significant changes were observed in the levels of activated B cells (CD19+CD38+) between times (PD-0 yr = 16.21 ± 6.70; PD-1 yr = 12.93 ± 4.52; PD-2 yr = 13.70 ± 5.18). Nevertheless, when the patients were stratified by treatment type, we observed that patients treated with a levodopa/pramipexole combination showed decreased levels of IL-10+ plasma cells after 2 years of treatment (Fig. 4B).

Finally, significant changes in peripheral immunity in individual patients throughout the period under study are shown in the Additional File 7: Figure S2.

## Discussion

In this work we analyzed immune cell populations in the peripheral blood of PD patients. PD patients were sampled 1 and 2 years after the start of the treatment. The immune response in PD patients showed changes in several cell subpopulations rarely studied before. The analysis of naive and memory CD4 and CD8 T cells demonstrated a decrease in naive/central memory CD4 T cells in PD patients throughout the period under study. We also found decreased levels of naive/central memory CD8 T cells 1 year after inclusion. These results are consistent with previous reports, in which decreased levels of naive T cells have been observed in PD patients [13, 26], suggesting that the decrease in naive T cells is a particular feature of PD.

On the other hand, increased levels of effector CD4 T cells were observed after 1 and 2 years of treatment, along with increased frequencies of activated CD4 T cells after 2 years with respect to untreated PD patients and after 1 year of treatment. It should be noted that while naive cell levels decreased, those of effector and activated cells increased. This fact has been associated with chronic cell activation related to aging and with viral infections like HIV [27–31]. However, this increase in the levels of activated and effector cells could also be the result of the chronic inflammation that characterizes the disease [27, 28]. Furthermore, it has been proposed that alterations in the balance of naive and effector/memory T cells could be related to the progression of PD [27]. We found a negative correlation between the levels of naive/central memory CD4 T cells with the H&Y score, suggesting that increased levels of these cells may favor a slower progression of the disease. In contrast, a positive correlation between effector T cell levels and the H&Y scale could indicate that effector cells play a role in the progression of the disease.

To our knowledge, CD8 Tc cells had not been previously characterized in PD patients. A decrease in the levels of Tc1 and Tc2 cells was found in PD-2 yr patients with respect to PD-1 yr and PD-0 yr. Tc1 cells are known to contribute to clear intracellular pathogens by producing cytotoxic molecules such as perforins and granzymes, as well as IFN-γ and TNF-α [32]. On the other hand, Tc2 cells exhibit a lower cytotoxic effect and are characterized by the production of cytokines like IL-5 and IL-13 [32]. The decrease in the levels of Tc1 and Tc2 cells after 2 years of follow-up suggests that neither of these profiles participates significantly in the inflammatory process in PD. A negative correlation between the levels of Tc1 and Tc2 cells with the H&Y scale score was observed in patients who only received treatment with levodopa during the follow-up period. This suggests that levodopa could affect Tc cells; however, it is currently unknown whether these cells express dopamine receptors. Therefore, evaluating the presence of dopamine receptors in these cells would help us to better understand the role of Tc cells in PD. In future studies, it would be important to determine the cause of the decrease in Tc1 and Tc2 cells.

It is remarkable that in the second year of follow-up, the decreased levels of Tc1 and Tc2 cells coincided with increased levels of Tc17 cells after 2 years of treatment. In vitro studies have shown that levodopa could favor the generation of Th17 cells from naïve T cells [33]. Thus, we can suggest that PD treatment could have a significant influence on the changes in Tc cell profiles in PD patients. The increased levels of Tc17 cells in PD patients could impact on their inflammatory response. Because the main function of Tc17 cells is the production of cytokines (IL-17A, IL-21, IL-23) [32, 34], it is possible that a dysregulation in cytokine availability in the brain of PD is contributing to the neurodegenerative process. For instance, the administration of IL-21 before the induction of experimental autoimmune encephalomyelitis promotes the activation of T and NK cells in the periphery and contributes to the severity of the disease. These results suggest that IL-21 could be a relevant factor in promoting PD [35, 36]. Several studies in vitro have shown that an overexpression of IL-17A is sufficient to generate a neuroinflammatory response in glial cells. Studies in brain tissue of patients with multiple sclerosis have found infiltration of CD4 and CD8 T cells, and both cell types have shown positivity to IL-17 [37].

Decreased levels of NKT cells were previously observed by other groups [38]. There is evidence that NKT cells have pro-inflammatory functions; this fact could be related to our finding of a positive correlation between the levels of NKT cells and the MDS-UPDRS scale score. Based on our observation that NKT decreased in PD patients, it is possible that these cells may not participate in the inflammatory process and may be related to dopaminergic treatment instead. The presence of dopamine receptors (DR) in NKT cells has not been established. Moreover, the expression of DR on CD8 T cells leads us to think that NKT cells could also express DR, since NKT cells express the CD8αα+ and α+β+ chains. Likewise, the presence of CD8 cells and their contribution to the inflammatory processes of the CNS have been observed [39–41]. Therefore, NKT cells could participate in the neurodegeneration processes in the central nervous system. However, while PD treatment could promote a reduction of these cell populations, this effect has not been demonstrated.

NKT cells are characterized by their ability to recognize lipid antigens. In multiple sclerosis patients, a response is generated against myelin in neuronal axons. Myelin is a molecule made up mainly by lipoproteins, which triggers the response and activation of NKT cells. In PD, alpha-synuclein could trigger an antigenic response like that of myelin [39, 42–44]. Due to its function as part of the cell membrane, alpha-synuclein has amphipathic properties. This allows it to associate with the lipid bilayer in the plasma membrane [43, 44]. This way, the structure of alpha-synuclein could also be recognized by NKT cells, triggering their activation and effector functions, which in turn could contribute to the neuroinflammatory process.

Decreased levels of plasma cells were found 2 years after treatment start with respect to year 1, along with decreased levels of Lip-AP B cells 1 and 2 years after with respect to the levels at inclusion time. This fact could be related to the expression of Blip-1 in Lip-AP B cells, which can regulate the production of IL-10 by B and plasma cells, as well as control its generation and differentiation into plasma cells [45]. Therefore, the decreased levels of Lip-AP B cells could be leading to decreased levels of plasma cells.

Changes in the immune response of the patients included in this study were previously evaluated, as reported in Álvarez-Luquín et al. [46] and Espinoza-Cardenas et al. [47]. In those works, other cell phenotypes were evaluated, including T helper populations. This study is aimed to expand our knowledge on the changes in the immune response observed in PD patients.

The age of patients is an important trait to consider, since various changes in the immune response have been associated with aging [27, 28, 48–50]. We found a negative correlation between naive CD4 T cells and age in the PD-1 yr group. This could be due to the thymus involution frequently observed in elderly patients, who show a lower production of naive T cells [27, 28] At the same time, we found a positive correlation between effector cell levels and age in the PD-1 yr group. This suggests an accumulation of memory cells and senescent effectors in the peripheral blood of elderly patients [27, 28].

We also observed a positive correlation between the levels of plasma cells and age in PD patients. Other studies have suggested that long-lived plasma cells could accumulate with age, and older patients show increased levels of this cell population [51]. Thus, our findings suggest that not only PD influences the observed changes in immune cell populations, but aging could also play a role [27, 28, 48–50].

It should be mentioned that this study has various limitations, being the sample size and the high patient drop-off rate the most important ones. More studies of this type in a larger population would be required to obtain more reliable results. Another limitation was the analysis of populations named as “like”. We decided to analyze these phenotypes due to the expression of activation markers that could suggest they play a role in the inflammatory stage of PD.

## Conclusion

Our results showed a decrease in the levels of naive/central memory CD4 T cells, CD8 T cells, plasma cells, and Lip-AP B cells, along with increased levels of effector and activated CD4 T cells and Tc17 cells in PD patients. These alterations could lead to an enhanced inflammatory response and favor the progression of PD. On the other hand, we found a decrease in the levels of NKT cells. These changes could promote PD progression.

The results herein reported show that age and treatment are key factors that can modulate and change the immune response in PD patients. Therefore, it is crucial to deepen our knowledge on those factors and their effects, to improve the management of the disease.

## Supplementary Information

**Additional file 1:Table S1.** Cell populations analyzed by flow cytometry.

**Additional file 2:Table S2.** Phenotypes of CD4, CD8 T, and CD19 cells analysed.

**Additional file 3:Figure S1.** Analysis strategy for CD4, CD8, and CD19 cells. In all cases, a singlet region, named as R1, was selected. Starting from this region, a forward vs scatter dot plot was created, defining a new region for the targeted cells (lymphocytes or plasma cells) which was named as R2. A. CD4 T cells. starting from R2, a new CD4+ window was created and named as R3. From R3, a new CD25 vs. CD127 window was created; CD4+CD25−CD127− cells were defined as effector CD4 T cells, while CD4+CD25−CD127+ cells were defined as naïve/central memory CD4 T cells. In another sample tube, starting from R2, a CD4 vs. CD25 window was created; from the double-positive region, a new IL-10 vs. TGF-b window was created; CD4+CD25+IL-10−TGF-b− cells were defined as activated CD4 non-Tregs. B. CD8 T cells. Starting from R2, a new CD8 vs. CD45RO window was created; from CD8+CD45RO−, a new IL-10 vs. CCR7 window was created; CD8+CD45RO-CCR7+IL-10- cells were defined as naïve CD8 non-Tregs. From the CD8+CD45RO+ cells, three windows of CCR7 vs. IL-10 were generated; CD8+ CD45RO+ CCR7− cells were defined as effector memory CD8; CD8+CD45RO+CCR7+ cells were defined as central memory CD8, while CD8+ CD45RO+CCR7-IL-10− cells were defined as effector memory CD8 non-Tregs. In another sample tube, starting from R2, a new CD8 vs. CD28 window was created; double-positive cells were defined as like-activated CD8 T cells. From R2, a new CD8 vs. CD161 window was created; CD8+CD161HI cells were defined as invariant, mucosa-associated-like cells (MAIT-like). From R2, a new CD8+ window was created and named as R3. From R3, a new CD56 vs. CD161 window was created; CD8+CD56+CD161+ cells were defined as NKT cells. C. CD8 T cytotoxic cells. The analysis of Tc cells was made in different tubes (one tube for each population). In all three cases, the gating strategy followed the same pattern. Starting from R2, a new CD8+ window was created and named as R3. From R3, a new window considering transcription factor vs. the corresponding cytokine was created; the following phenotypes were defined with this strategy: Tc1 (CD8+TBET+IFN-γ+ or CD8+TBET+TNF-α+), Tc2 (CD8+GATA-3+IL-13+ or CD8+GATA-3+IL-4+), and Tc17 (CD8+ROR- γ +IL-17+). D. B cells. Starting from R2, a new CD19− region was generated and named as R4. Starting from R4, a new CD138 vs. CD38 window was generated; CD19−CD138+ CD38+ cells were defined as plasma cells. From R2, a new CD19 vs. CD138 window was generated; CD138+ cells were selected, and the expression of IL-10 was measured in this region; CD138+IL-10+ were defined as L-10+ plasma cells. Also, from R2, a new CD19 vs. CD38 window was created; double-positive cells were defined as activated B cells. In a different sample tube, starting from R2, a new CD19 vs. CD1d window was generated; CD19+CD1d+ cells were defined as lipid-antigen presenting (Lip-AP) B cells.

**Additional file 4: Table S3.** Levels of population markers. Comparison between patients and controls and patients with themselves at different times.

**Additional file 5: Table S4.** Changes in immune cell populations associated with age.

**Additional file 6: Table S5.** Changes in T cytotoxic phenotypes in patients and the control group.

**Additional file 7: Figure S2.** Changes in immune populations during follow-up.

## Data Availability

The datasets used and/or analyzed in this study are available from the corresponding author on reasonable request.

## Abbreviations

ACD: Acid-citrate-dextrose
Bregs: Regulatory B cells
CD: Cluster of differentiation
CD8regs: Regulatory CD8 T cells
CNS: Central nervous system
COMT: Catechol-O-methyltransferase
DCs: Dendritic cells
DR: Dopamine receptor
HBP: High blood pressure
HIV: Human immunodeficiency virus
H&Y: Hoehn and Yahr
HLA: Human leukocyte antigen complex
IFN-γ: Interferon gamma
IL: Interleukin
INNN: Instituto Nacional de Neurología y Neurocirugía
MAIT: Mucosal-associated invariant T cells
MAO: Monoamine oxidase
NF-κB: Nuclear factor-kappa B
NKs: Natural killer cells
NKTs: Natural killer T cells
NT-proCNP: N-Terminal pro-C-type natriuretic peptide
PBMCs: Peripheral blood mononuclear cells
PBS: Phosphate buffer solution
PD: Parkinson’s disease
PD-L1: Programmed death-ligand 1
SNpc: Substance nigra pars compacta
Tc: T cytotoxic
TGF-β: Transforming growth factor beta
Th: T helper
TNF-α: Tumor necrosis factor alpha
Tr1: Type-1 regulatory T cells
Tregs: Regulatory T cells
UK PDSBB: United Kingdom Parkinson’s Disease Society Brain Bank
MDS-UPDRs: Movement disorder society-sponsored revision of the unified Parkinson’s disease rating scale
