# Supplementary material for: Increased Tc17 cell levels and imbalance of naïve/effector immune response in Parkinson’s disease patients in a two-year follow-up: a case control study
Source: J Transl Med. 2021 Sep 6;19:378. doi: 10.1186/s12967-021-03055-2 (PMC8422782; doi:10.1186/s12967-021-03055-2)
Supplement: Supplementary file 1 — Additional file 1:Table S1. Cell populations analyzed by flow cytometry. [file 12967_2021_3055_MOESM1_ESM.pdf]

Supplementary Table 1. Cell populations analyzed by flow cytometry

Cell population	Isotype	Antibody	Vol. Ab (µL)	#Reference
CD4 T Cells	mouse IgG1k FITC	CD127 FITC[a]	3.75	11-1278-42
	rat IgG2ak PE	IL-10 PE[a]*	5	12-7108-82
	mouse IgG1k PerCP Cy5.5	TGF-β PerCP Cy5.5[d]*	5	349612
	mouse IgG1k APC	CD25 APC[b]	5	340939
	mouse IgG1k APC Cy7	CD4 APC Cy7[b]	2.5	557871
CD8 T Cells	mouse IgG1k FITC	CD56 FITC[a]	5	11-0566-42
	mouse IgG1k FITC	IFN-γ FITC[a]*	7.5	BMS107FI
	rat IgG2ak FITC	CCR7 FITC[a]	5	11-1979-42
	mouse IgG1k FITC	IL-13 FITC[a]*	5	11-7139-42
	mouse IgG1k FITC	IL-17α FITC[a]*	3.75	11-7179-42
	rat IgG2ak PE	IL-10 PE[a]*	5	12-7108-82
	mouse IgG1k PE	Tbet PE[a]*	3.75	12-5825-82
	mouse IgG1k PE	IL-4 PE[a]*	5	12-7049-42
	rat IgG2a PE	ROR-γ PE[a]*	3.75	12-6988-82
	mouse IgG2ak PerCP	CD45RO PerCP[c]	5	MHCD45RO31
	IgG1 PerCP Cy5.5	CD161 PerCP[a]	5	45-1619-42
	mouse IgG1k PerCP Cy5.5	TNF-α PerCP Cy5.5[a]*	5	560679
	rat IgG2bk PerCP eFluor 710	GATA-3 PerCP[a]*	5	46-9966-42
	mouse IgG1k APC	CD8 APC[a]	2.5	17-0088-42
	mouse IgG1k APC H7	CD28 APC H7[b]	3.75	561368
B Cells	mouse IgG1k FITC	CD138 FITC[a]	5	11-1389-42
	rat IgG2ak PE	IL-10 PE[a]*	5	12-7108-82
	mouse PerCP eFluor 710	CD1d PerCP eFluor 710[b]	5	46-0016-42
	mouse IgG1k APC	CD38 APC[a]	3.75	17-0389-42
	mouse IgG1k APC Cy7	CD19 APC Cy7[b]	2.5	557791

The antibodies used in this study were purchased from eBioscience (Waltham, MA, USA) ([a]), BD (Franklin Lakes, NJ, USA) ([b]), Invitrogen (Waltham, MA, USA) ([c]), and Biolegend (San Diego, CA, USA) ([d]); *intracellular antibodies.
