# Supplementary material for: Increased Tc17 cell levels and imbalance of naïve/effector immune response in Parkinson’s disease patients in a two-year follow-up: a case control study
Source: J Transl Med. 2021 Sep 6;19:378. doi: 10.1186/s12967-021-03055-2 (PMC8422782; doi:10.1186/s12967-021-03055-2)
Supplement: Supplementary file 2 — Additional file 2:Table S2. Phenotypes of CD4, CD8 T, and CD19 cells analysed. [file 12967_2021_3055_MOESM2_ESM.pdf]

Table 2. Phenotypes of CD4, CD8 T, and CD19 cells analyzed

Phenotype	Name
**CD4 T cells**	
CD4+CD25−CD127−	Effector CD4 T cells[1]
CD4+CD25+IL-10-TGF-β−	Activated CD4 non-Tregs
CD4+CD25-CD127+	Naive/central memory CD4 T cells[1]
**CD8 T cells**	
CD8+CD28+	Activated-like CD8 T cells[2]
CD8+CCR7−CD45RO+	Effector memory CD8 T cells[3]
CD8+CCR7+CD45O+	Central memory CD8 T cells[3]
CD8+CCR7−CD45RO+IL-10−	Effector memory CD8 non-Tregs
CD8+CCR7+CD45RO-IL-10−	Naive CD8 non-Tregs
CD8+TBET+IFN-γ+	Tc1 cells[4]
CD8+TBET+TNF-α+	Tc1 cells[4]
CD8+GATA3+IL-4+	Tc2 cells[4]
CD8+GATA3+IL-13+	Tc2 cells[4]
CD8+ROR-γ+IL-17+	Tc17 cells[4]
CD8+ROR-γ+IL-6+	Tc17 cells[4]
CD8+CD56+CD161+	NKT cells[5]
CD8+CD161^{HI}	MAIT-like [6]
**B cells**	
CD19-CD138+CD38+	Plasma cells[7]
CD138+IL-10+	IL-10+ Plasma cells
CD19+CD1d+	Lip AP B cells
CD19+CD38+	Activated B cells[8]

Mucosal associated invariant T cells (MAIT); Lipid-antigen presenting (Lip AP);

Bibliography

1. Dunham RM, Cervasi B, Brenchley JM, Albrecht H, Weintrob A, Sumpter B, et al. CD127 and CD25 Expression Defines CD4 + T Cell Subsets That Are Differentially Depleted during HIV Infection . The Journal of Immunology. 2008;180:5582–92.

2. Landay AL, Mackewicz CE, Levy JA. An activated CD8+ T cell phenotype correlates with anti-HIV activity and asymptomatic clinical status. Clinical immunology and immunopathology. United States; 1993;69:106–16.

3. Haegele KF, Stueckle CA, Malin J-P, Sindern E. Increase of CD8+ T-effector memory cells in peripheral blood of patients with relapsing-remitting multiple sclerosis compared to healthy controls. Journal of neuroimmunology. Netherlands; 2007;183:168–74.

4. Annunziato F, Romagnani C, Romagnani S. The 3 major types of innate and adaptive cell-mediated effector immunity. Journal of Allergy and Clinical Immunology [Internet]. Elsevier Ltd; 2015;135:626–35. Available from: http://dx.doi.org/10.1016/j.jaci.2014.11.001

5. Ohkawa T, Seki S, Dobashi H, Koike Y, Habu Y, Ami K, et al. Systematic characterization of human CD8+ T cells with natural killer cell markers in comparison with natural killer cells and normal CD8+ T cells. Immunology. 2001;103:281–90.

6. Fergusson JR, Fleming VM, Klenerman P. CD161-expressing human T cells. Frontiers in Immunology. 2011;2:1–7.

7. Garimalla S, Nguyen DC, Halliley JL, Tipton C, Rosenberg AF, Fucile CF, et al. Differential transcriptome and development of human peripheral plasma cell subsets. JCI insight [Internet]. American Society for Clinical Investigation; 2019;4:e126732. Available from: https://pubmed.ncbi.nlm.nih.gov/31045577

8. Liu Z, Zeng W, Huang X, Wang S, Zheng J, Pan M, et al. Peripheral CD19(hi) B cells exhibit activated phenotype and functionality in promoting IgG and IgM production in human autoimmune diseases. Scientific reports. 2017;7:13921.
