# Supplementary material for: Increased Tc17 cell levels and imbalance of naïve/effector immune response in Parkinson’s disease patients in a two-year follow-up: a case control study
Source: J Transl Med. 2021 Sep 6;19:378. doi: 10.1186/s12967-021-03055-2 (PMC8422782; doi:10.1186/s12967-021-03055-2)
Supplement: Supplementary file 3 — Additional file 3:Figure S1. Analysis strategy for CD4, CD8, and CD19 cells. In all cases, a singlet region, named as R1, was selected. Starting from this region, a forward vs scatter dot plot was created, defining a new region for the targeted cells (lymphocytes or plasma cells) which was named as R2. A. CD4 T cells. starting from R2, a new CD4+ window was created and named as R3. From R3, a new CD25 vs. CD127 window was created; CD4+CD25−CD127− cells were defined as effector CD4 T cells, while CD4+CD25−CD127+ cells were defined as naïve/central memory CD4 T cells. In another sample tube, starting from R2, a CD4 vs. CD25 window was created; from the double-positive region, a new IL-10 vs. TGF-b window was created; CD4+CD25+IL-10−TGF-b− cells were defined as activated CD4 non-Tregs. B. CD8 T cells. Starting from R2, a new CD8 vs. CD45RO window was created; from CD8+CD45RO−, a new IL-10 vs. CCR7 window was created; CD8+CD45RO-CCR7+IL-10- cells were defined as naïve CD8 non-Tregs. From the CD8+CD45RO+ cells, three windows of CCR7 vs. IL-10 were generated; CD8+ CD45RO+ CCR7− cells were defined as effector memory CD8; CD8+CD45RO+CCR7+ cells were defined as central memory CD8, while CD8+ CD45RO+CCR7-IL-10− cells were defined as effector memory CD8 non-Tregs. In another sample tube, starting from R2, a new CD8 vs. CD28 window was created; double-positive cells were defined as like-activated CD8 T cells. From R2, a new CD8 vs. CD161 window was created; CD8+CD161HI cells were defined as invariant, mucosa-associated-like cells (MAIT-like). From R2, a new CD8+ window was created and named as R3. From R3, a new CD56 vs. CD161 window was created; CD8+CD56+CD161+ cells were defined as NKT cells. C. CD8 T cytotoxic cells. The analysis of Tc cells was made in different tubes (one tube for each population). In all three cases, the gating strategy followed the same pattern. Starting from R2, a new CD8+ window was created and named as R3. From R3, a new window considering transcripti [file 12967_2021_3055_MOESM3_ESM.pdf]

Supplementary Figure 1. Analysis strategy for CD4, CD8, and CD19 cells. In all cases, a singlet region, named as R1, was selected. Starting from this region, a forward vs scatter dot plot was created, defining a new region for the targeted cells (lymphocytes or plasma cells) which was named as R2. **A. CD4 T cells.** starting from R2, a new CD4+ window was created and named as R3. From R3, a new CD25 vs. CD127 window was created; CD4+CD25−CD127− cells were defined as effector CD4 T cells, while CD4+CD25−CD127+ cells were defined as naïve/central memory CD4 T cells. In another sample tube, starting from R2, a CD4 vs. CD25 window was created; from the double-positive region, a new IL-10 vs. TGF-b window was created; CD4+CD25+IL-10−TGF-b− cells were defined as activated CD4 non-Tregs. **B. CD8 T cells.** Starting from R2, a new CD8 vs. CD45RO window was created; from CD8+CD45RO−, a new IL-10 vs. CCR7 window was created; CD8+CD45RO-CCR7+IL-10- cells were defined as naïve CD8 non-Tregs. From the CD8+CD45RO+ cells, three windows of CCR7 vs. IL-10 were generated; CD8+ CD45RO+ CCR7− cells were defined as effector memory CD8; CD8+CD45RO+CCR7+ cells were defined as central memory CD8, while CD8+ CD45RO+CCR7-IL-10− cells were defined as effector memory CD8 non-Tregs. In another sample tube, starting from R2, a new CD8 vs. CD28 window was created; double-positive cells were defined as like-activated CD8 T cells. From R2, a new CD8 vs. CD161 window was created; CD8+CD161^{HI} cells were defined as invariant, mucosa-associated-like cells (MAIT-like). From R2, a new CD8+ window was created and named as R3. From R3, a new CD56 vs. CD161 window was created; CD8+CD56+CD161+ cells were defined as NKT cells. **C. CD8 T cytotoxic cells.** The analysis of Tc cells was made in different tubes (one tube for each population). In all three cases, the gating strategy followed the same pattern. Starting from R2, a new CD8+ window was created and named as R3. From R3, a new window considering transcription factor vs. the corresponding cytokine was created; the following phenotypes were defined with this strategy: Tc1 (CD8+TBET+IFN-γ+ or CD8+TBET+TNF-α+), Tc2 (CD8+GATA-3+IL-13+ or CD8+GATA-3+IL-4+), and Tc17 (CD8+ROR-γ+IL-17+). **D. B cells.** Starting from R2, a new CD19− region was generated and named as R4. Starting from R4, a new CD138 vs. CD38 window was generated; CD19−CD138+ CD38+ cells were defined as plasma cells. From R2, a new CD19 vs. CD138 window was generated; CD138+ cells were selected, and the expression of IL-10 was measured in this region;

CD138+IL-10+ were defined as L-10+ plasma cells. Also, from R2, a new CD19 vs. CD38 window was created; double-positive cells were defined as activated B cells. In a different sample tube, starting from R2, a new CD19 vs. CD1d window was generated; CD19+CD1d+ cells were defined as lipid-antigen presenting (Lip-AP) B cells.

A. CD4 T cells

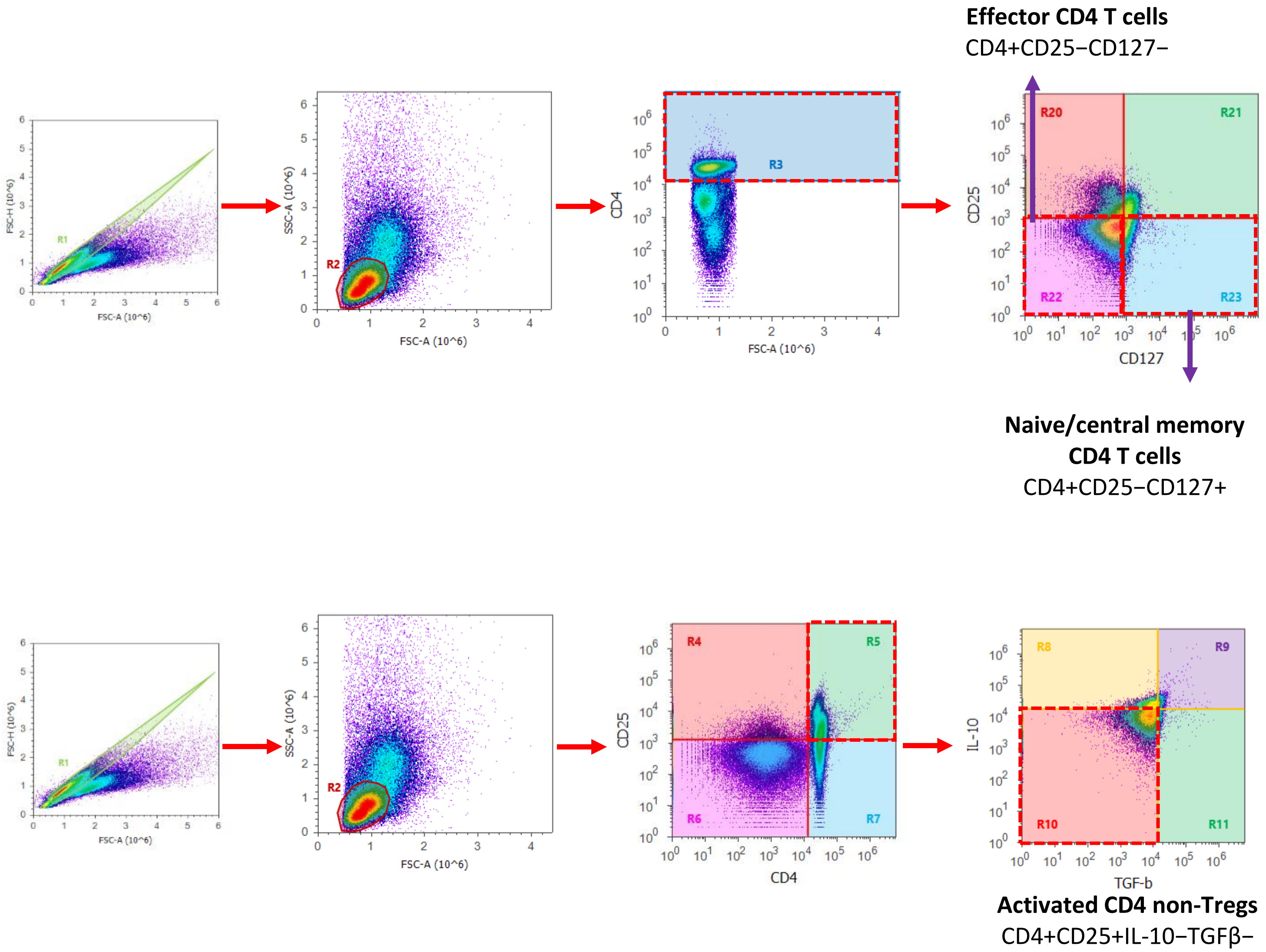

Effector CD4 T cells
CD4+CD25−CD127−
Naive/central memory
CD4 T cells
CD4+CD25−CD127+
Activated CD4 non-Tregs
CD4+CD25+IL-10−TGFβ−
R1
R2
R3
R4
R5
R6
R7
R8
R9
R10
R11
R20
R21
R22
R23
FSC-H (10^6)
FSC-A (10^6)
SSC-A (10^6)
CD4
CD25
CD127
IL-10
TGF-b

B. CD8 T cells

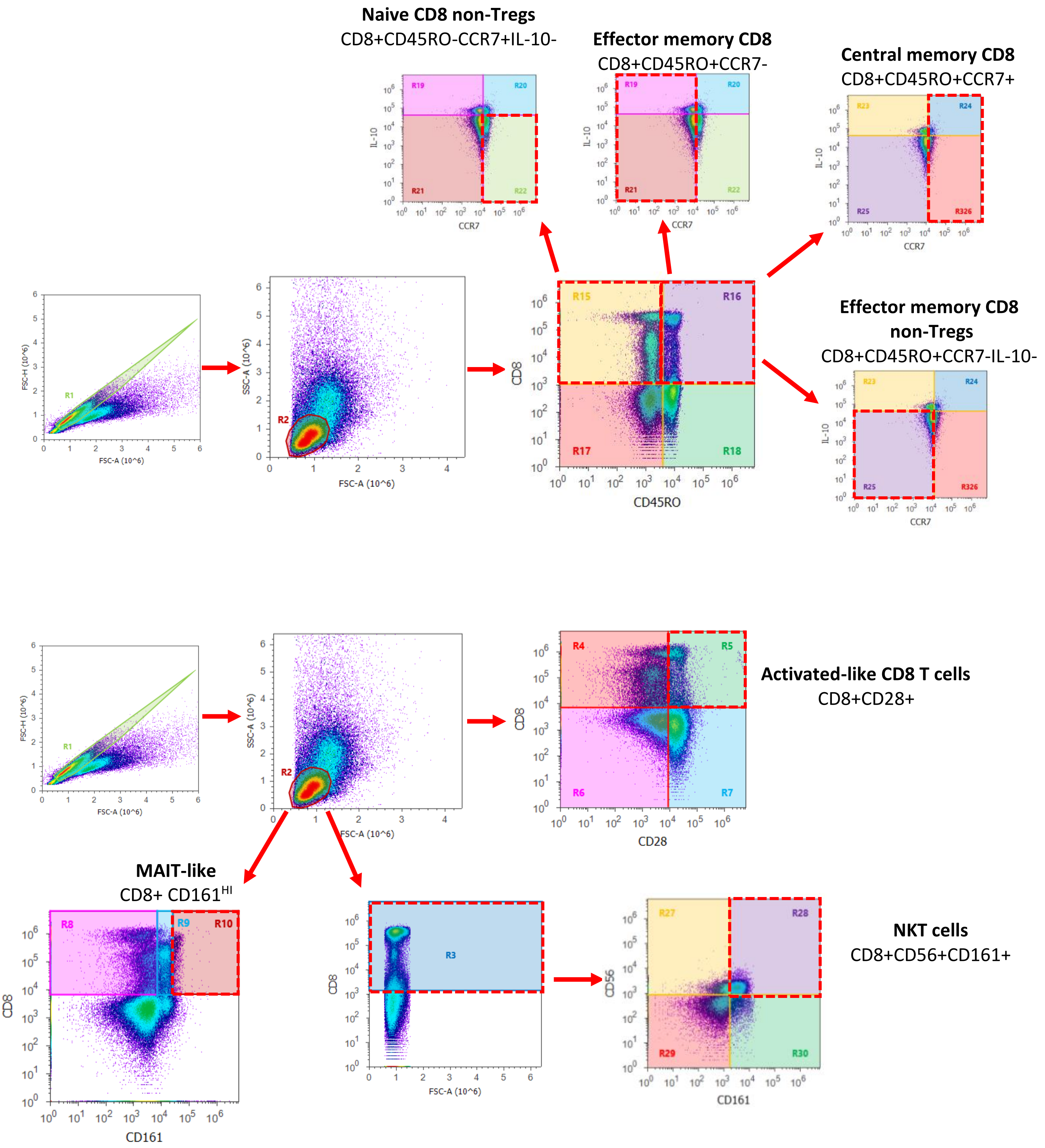

Naive CD8 non-Tregs
CD8+CD45RO-CCR7+IL-10-
Effector memory CD8
CD8+CD45RO+CCR7-
Central memory CD8
CD8+CD45RO+CCR7+
Effector memory CD8
non-Tregs
CD8+CD45RO+CCR7-IL-10-
FSC-H (10^6)
FSC-A (10^6)
SSC-A (10^6)
R1
R2
CD8
CD45RO
R15
R16
R17
R18
IL-10
CCR7
R19
R20
R21
R22
R23
R24
R25
R326
Activated-like CD8 T cells
CD8+CD28+
CD28
R4
R5
R6
R7
MAIT-like
CD8+ CD161HI
CD161
R8
R9
R10
R3
CD56
R27
R28
R29
R30
NKT cells
CD8+CD56+CD161+

C. CD8 T cytotoxic cells

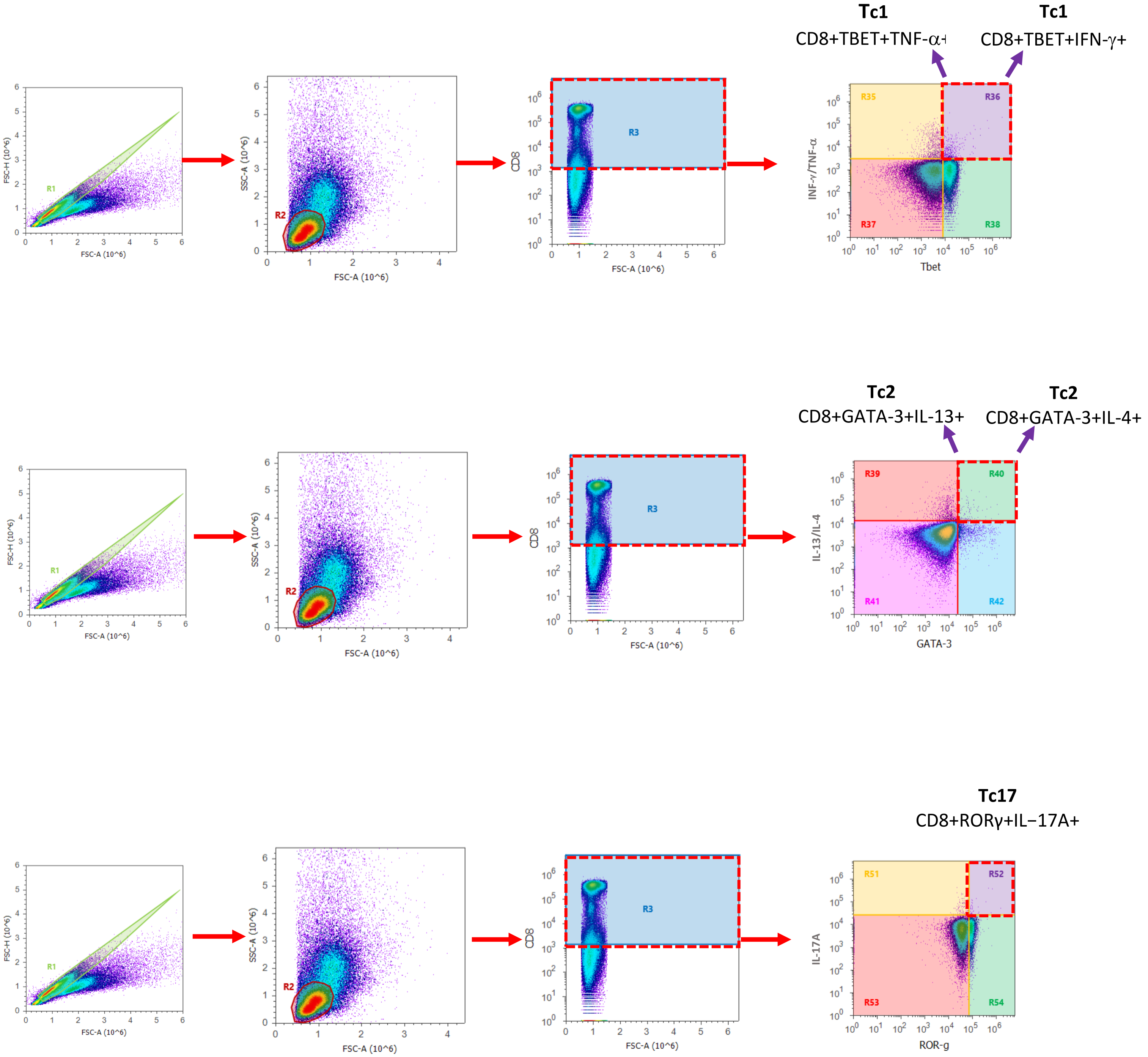

D. B cells

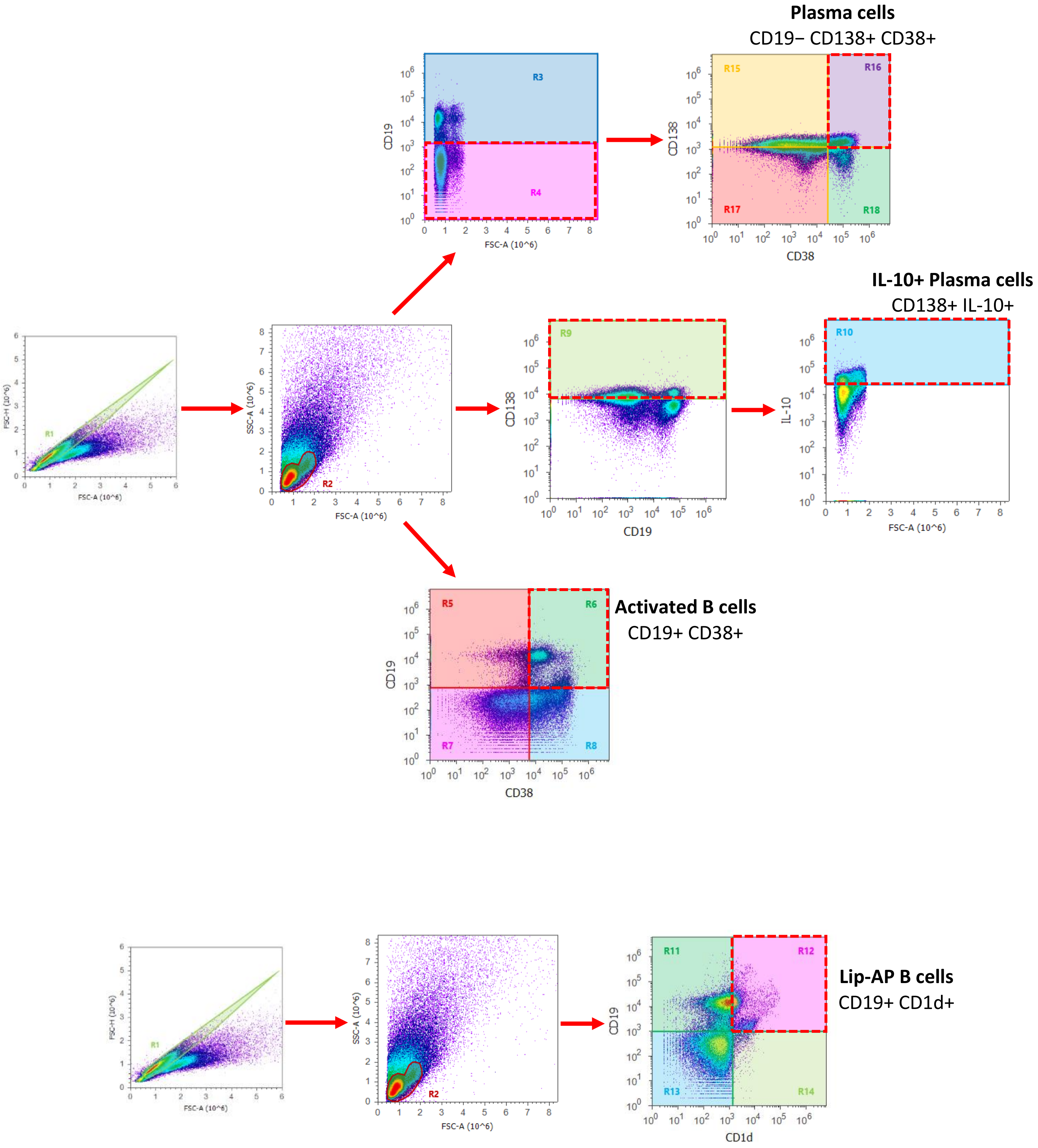

Plasma cells
CD19– CD138+ CD38+
IL-10+ Plasma cells
CD138+ IL-10+
Activated B cells
CD19+ CD38+
Lip-AP B cells
CD19+ CD1d+
R1
R2
R3
R4
R5
R6
R7
R8
R9
R10
R11
R12
R13
R14
R15
R16
R17
R18
FSC-H (10^6)
FSC-A (10^6)
SSC-A (10^6)
CD19
CD138
CD38
IL-10
CD1d
