# Supplementary material for: Increased Tc17 cell levels and imbalance of naïve/effector immune response in Parkinson’s disease patients in a two-year follow-up: a case control study
Source: J Transl Med. 2021 Sep 6;19:378. doi: 10.1186/s12967-021-03055-2 (PMC8422782; doi:10.1186/s12967-021-03055-2)
Supplement: Supplementary file 4 — Additional file 4: Table S3. Levels of population markers. Comparison between patients and controls and patients with themselves at different times. [file 12967_2021_3055_MOESM4_ESM.pdf]

Supplementary Table 3. Levels of population markers. Comparison between patients and controls and patients with themselves at different times.

	Controls[¢]	PD-0yr[¢]	PD-1yr[¢]	PD-2yr[¢]	PD-0yr vs PD-1yr	PD-0yr vs PD-2yr	PD-1yr vs PD-2yr
CD4	39.05[29.03-45.04]	32.09[26.25-37.82] *P* = 0.0115[+]	37.42[27.41-44.00] *P* = 0.7451	40.06[29.74-44.31] *P* = 0.9770	0.0395*****	0.4922	0.6953
CD8	33.75[25.96-38.63]	35.12[30.38-39.76] *P* = 0.3770	30.55[29.07-34.33] *P* = 0.5352	33.02[28.50-39.35] *P* = 0.9627	0.1324	0.9219	0.9219
CD19	18.04[13.19-21.75]	16.77[13.08-22.68] *P* = 0.8429	15.50[12.86-18.72] *P* = 0.1972	19.13[14.87-23.68] *P* = 0.6629	0.2247	0.7695	0.6250

[¢] Data are reported as median and interquartile range [IQR]. Differences between patients and controls are shown below the median for each group. Differences between patients and themselves during the follow-up are shown in the tree last columns. PD-0yr (untreated Parkinson’s disease patients), PD-1yr (patients treated for one year), PD-2yr (patients treated for two years). (*) indicate significant differences between patient groups. (*) indicates significant differences between patients and control subjects. *P* < 0.05 (*); *P* < 0.005 (**); *P* < 0.0005 (***).
