# Supplementary material for: Increased Tc17 cell levels and imbalance of naïve/effector immune response in Parkinson’s disease patients in a two-year follow-up: a case control study
Source: J Transl Med. 2021 Sep 6;19:378. doi: 10.1186/s12967-021-03055-2 (PMC8422782; doi:10.1186/s12967-021-03055-2)
Supplement: Supplementary file 5 — Additional file 5: Table S4. Changes in immune cell populations associated with age. [file 12967_2021_3055_MOESM5_ESM.pdf]

Supplementary Table 4. Changes in immune cell populations associated with age.

	Control		PD-0yr		PD-1yr		PD-2yr	
	P	*r*	*P*	*r*	*P*	*r*	*P*	*r*
Effector CD4 T cells CD4+CD25−CD127-	0.295	0.253	0.810	0.046	0.004**	0.599	0.578	−0.201
Naive/central memory CD4 T cells CD4+CD25−CD127+	0.184	0.253	0.703	−0.072	0.039*	−0.453	0.210	0.434
Plasma cells CD19−CD138+CD38+	0.071	−0.411	0.012*	0.461	0.754	−0.075	0.957	−0.019

Correlation between age and cell population levels. PD-0yr (untreated Parkinson's disease patients), PD-1yr (patients treated for one year), PD-2yr (patients treated for two years). $P < 0.05$ (*); $P < 0.005$ (**).
