# Supplementary material for: Increased Tc17 cell levels and imbalance of naïve/effector immune response in Parkinson’s disease patients in a two-year follow-up: a case control study
Source: J Transl Med. 2021 Sep 6;19:378. doi: 10.1186/s12967-021-03055-2 (PMC8422782; doi:10.1186/s12967-021-03055-2)
Supplement: Supplementary file 6 — Additional file 6: Table S5. Changes in T cytotoxic phenotypes in patients and the control group. [file 12967_2021_3055_MOESM6_ESM.pdf]

Supplementary Table 5. Changes in T cytotoxic phenotypes in patients and the control group.

	Phenotype	Controls[¢]	PD-0yr[¢]	PD-1yr[¢]	PD-2yr[¢]	PD-0yr vs PD-1yr *P*-value	PD-0yr vs PD-2yr *P*-value	PD-1yr vs PD-2yr *P*-value
Tc1	$CD8^+$ $TBET^+$	55.53[46.88-72.16]	65.31[52.84-73.80] *P*=0.200	62.90[55.44-73.38] *P*=0.176	71.68[52.95-79.35] *P*=0.156	0.973	0.432	0.769
	$CD8^+$ $TBET^+$ IFN-γ^+	0.41[0.23-0.74]	0.40[0.25-0.74] *P*=0.102	0.21[0.07-0.36] *P*=0.802	0.11[0.07-0.14] *P*=0.024*	0.374	0.004**	0.009*
	$CD8^+$ $TBET^+$ TNF-α^+	0.51[0.22-1.019]	0.50[0.36-0.85] *P*=0.801	0.31[0.18-0.87] *P*=0.375	0.29[0.17-0.39] *P*=0.034*	0.926	0.064	0.064
Tc2	$CD8^+GATA3^+$	4.39[3.69-7.07]	3.08[2.32-5.12] *P*=0.937	2.73[1.39-4.42] *P*=0.198	2.26[1.52-3.74] *P*=0.206	0.036*	0.039*	0.064
	$CD8^+GATA3^+$ IL-13^+	0.30[0.11-0.48]	0.30[0.15-0.61] *P*=0.937	0.21[0.09-0.31] *P*=0.198	0.15[0.05-0.18] *P*=0.206	0.007*	0.027*	0.436
	$CD8^+GATA3^+$IL-4^+	0.20[0.09-0.58]	0.10[0.07-0.37] *P*=0.102	0.25[0.07-0.61] *P*=0.779	0.12[0.08-0.59] *P*=0.245	0.522	0.164	0.160
Tc17	$CD8^+$ROR-γ^+	29.81[24.26-38.73]	17.69[9.33-56.01] *P*=0.119	24.68[4.68-35.46] *P*=0.568	51.05[18.54-81.02] *P*=0.082	0.522	0.375	0.039*
	$CD8^+$ROR-γ^+IL-17^+	0.05[0.02-0.14]	0.07[0.03-0.16] *P*=0.427	0.03[0.01-0.04] *P*=0.009*	0.06[0.4-0.09] *P*=0.856	0.001**	0.078	0.250

[¢] Data are reported as median and interquartile range [IQR]. Differences between patients and controls are shown below the median for each group. Differences between patients and themselves during the follow-up are shown in the tree last columns. PD-0yr (untreated Parkinson's disease patients), PD-1yr (patients treated for one year), PD-2yr (patients treated for two years). (*) indicate significant differences between patient groups or between a patient group and the control group. $P < 0.05$ (*); $P < 0.005$ (**); $P < 0.0005$ (***).
