# Supplementary figures and images for: Increased Tc17 cell levels and imbalance of naïve/effector immune response in Parkinson’s disease patients in a two-year follow-up: a case control study
Source: J Transl Med. 2021 Sep 6;19:378. doi: 10.1186/s12967-021-03055-2 (PMC8422782; doi:10.1186/s12967-021-03055-2)

Supplementary Figure 2. Changes in immune populations during follow-up

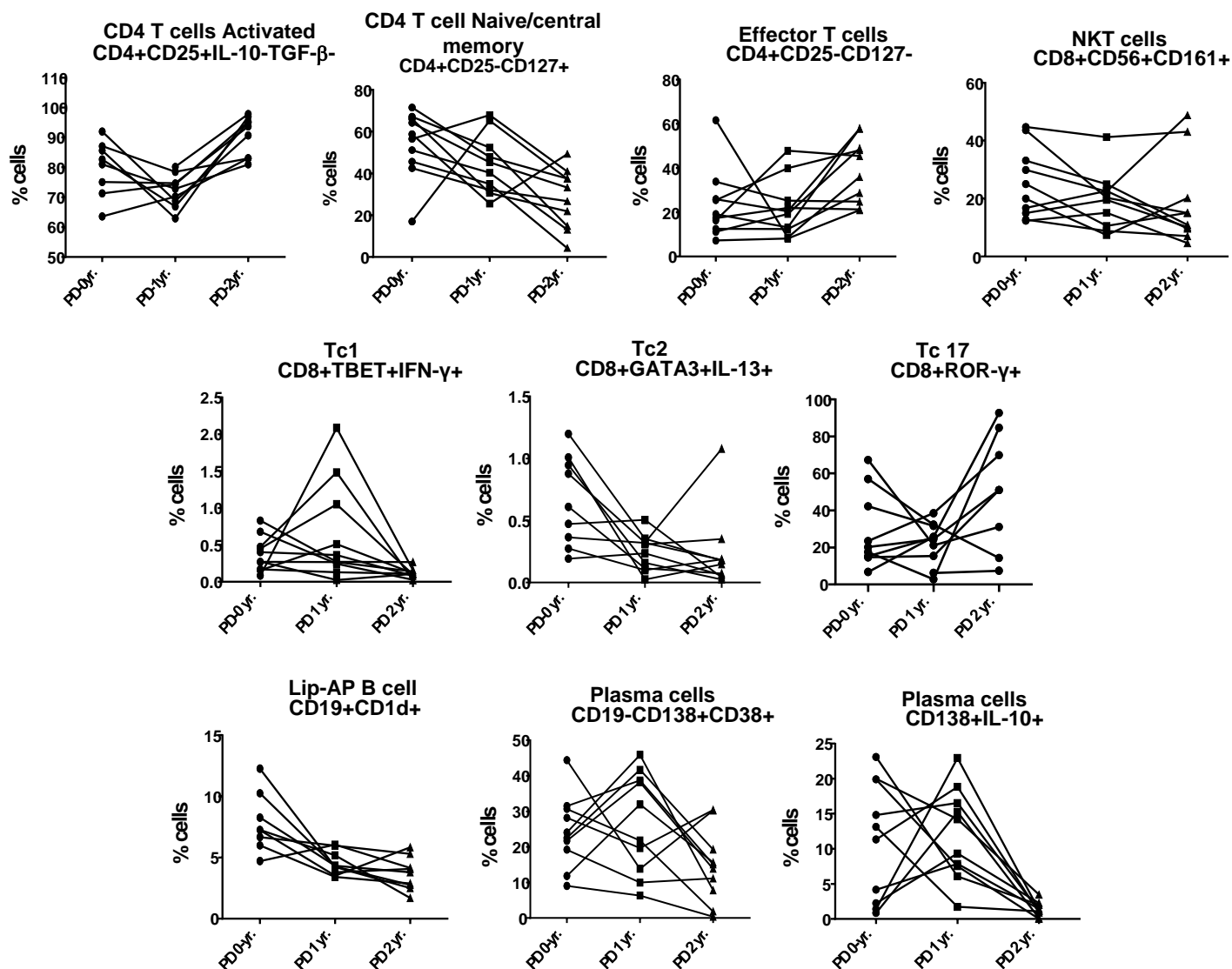

Supplement: Supplementary file 7 — Additional file 7: Figure S2. Changes in immune populations during follow-up. [file 12967_2021_3055_MOESM7_ESM.pdf]
